# Artificial Intelligence in Healthcare: Perceptions among Older Adults in Northern Ontario, Canada

**DOI:** 10.1002/alz70860_106964

**Published:** 2025-12-23

**Authors:** Lucy Shrestha, Hom Lal Shrestha

**Affiliations:** ^1^ School of Kinesiology and Health Sciences, Laurentian University, Sudbury, ON, Canada; ^2^ Faculty of Health, York University, Toronto, ON, Canada

## Abstract

**Background:**

Globally, the size and proportion of older adults are growing at an unprecedented rate. Given the rising incidence of health complications among older adults, it is critical to understand opportunities for improving the delivery of health care. Artificial intelligence (AI) is a promising technology that many argue has the potential to improve patient care, particularly by supporting and assisting older adults in diagnosing, managing, and treating health conditions. Although there have been significant studies on AI innovations in health care, there is an overall lack of literature on patient perceptions of the implementation of AI, specifically in older adult populations. This study addresses this gap in research by engaging older adults and focusing on AI and healthy aging.

**Method:**

Utilizing an appreciative inquiry framework, the data collection consists of interviews with older adults to develop an understanding of how older adults perceive the use and consideration of AI in Northern Ontario, Canada. We analyze the findings using Braun and Clark's (2022) thematic analysis six‐step approach.

**Result:**

The findings from this study highlight how AI technologies can better support older adults in Northern Ontario. The study highlights identifying older adults' perceptions toward AI in healthcare settings, as well as the perceived barriers and facilitators to AI utilization in Adult Day Centers/Older Adults Centers and Retirement Homes. The study contributes to the development of specialized educational materials or training programs designed to raise awareness and practice, encourage advocacy, improve comfort levels with AI technologies, and ultimately enhance older adult patient care. The findings will be informed to policymakers and stakeholders for adjusting policy reform and analysis. This study shed light on how older adults feel toward AI, how they envision improvements, and what improvements could be made for future health practices using AI technologies.

**Conclusion:**

Overall, this research could help identify factors contributing to better AI‐enabled care of older adults, and it could shed light on how AI technologies could evolve to better meet the needs of older adults.

